# Strengthening stroke prevention and awareness in the Philippines: a conceptual framework

**DOI:** 10.3389/fneur.2023.1258821

**Published:** 2023-08-31

**Authors:** Lance Vincent C. Sese, Ma. Celina L. Guillermo

**Affiliations:** ^1^Nuffield Department of Primary Care Health Sciences, University of Oxford, Oxford, United Kingdom; ^2^Department for Continuing Education, University of Oxford, Oxford, United Kingdom; ^3^Department of Neurology, National Hospital for Neurology and Neurosurgery, University College London Hospitals NHS Foundation Trust, London, United Kingdom; ^4^Ateneo School of Medicine and Public Health, Don Eugenio Lopez Sr. Medical Complex, Pasig, Metro Manila, Philippines; ^5^ThinkWell Global, Binondo, Metro Manila, Philippines

**Keywords:** Philippines, stroke, cerebrovascular disease, patient education, prevention, public health

## Abstract

Stroke is a leading cause of mortality and disability worldwide, with low and middle-income countries bearing the greatest burden. This article focuses on stroke prevention and awareness in the Philippines, a country grappling with high stroke incidence and limited healthcare resources. The two-pronged approach presented by the authors aims to address the challenges of stroke care by combining community-based prevention and targeted public awareness campaigns. The community-based stroke prevention component involves personalized risk factor assessments and tailored interventions conducted at local health centers. By identifying modifiable risk factors such as hypertension, diabetes, smoking, and elevated cholesterol levels, healthcare professionals can provide targeted education and interventions to individuals at risk. Additionally, the decentralized targeted stroke awareness campaigns emphasize public education through culturally adapted materials, engagement with local stakeholders, and media campaigns. These initiatives seek to increase awareness of stroke symptoms and prompt presentation in medical facilities. By implementing this comprehensive approach, we aim to mitigate the burden of stroke in the Philippines, improve stroke outcomes, and raise public awareness about stroke recognition and prevention.

## 1. Introduction

**Figure 1:**
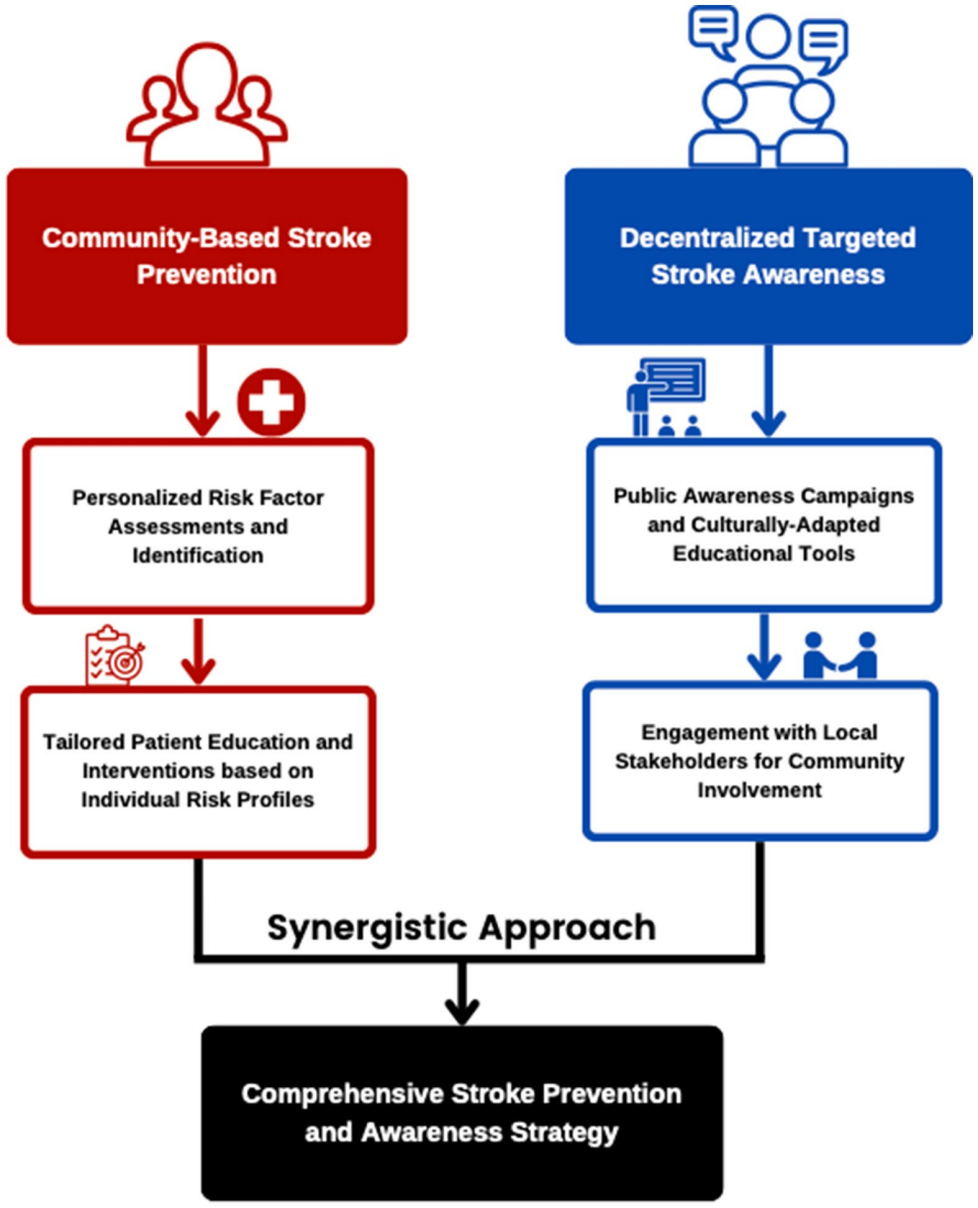
The figure shows the two-pronged complementary approach for stroke awareness and prevention. The community-based stroke prevention focuses on personalized risk factor assessments and tailored patient education and interventions. The decentralized targeted stroke awareness tackles public awareness campaigns, culturally adapted educational materials, and engagement with local stakeholders.

Stroke has always been part of the leading causes of mortality and disability worldwide, with low and middle-income countries suffering the burden the most (1). Globally, there were 13.7 million new strokes in 2016, with approximately 87% being ischemic strokes and an estimated 10–20% of these attributed to large vessel occlusion (LVO), yet less than 5% of acute ischemic stroke patients received intravenous thrombolysis (IVT) within the appropriate time frame, and fewer than 100,000 mechanical thrombectomies (MTs) were performed worldwide that year (1). Hence, addressing gaps in stroke care becomes a priority by bridging what is known and what is done (2). As one of the countries in the Southeast Asian region belonging to the lower-middle-income countries, the Philippines is no exception. In 2021 and 2022, stroke or cerebrovascular disease was the second leading cause of death in the country (3), with many Filipinos, particularly those in lower income brackets suffering from residual disabilities. From January to May 2022 alone, there was a recorded 21,602 deaths due to stroke (10.4% of total deaths), slowly trailing behind COVID-19 and cardiovascular-related deaths (3). Unfortunately, a significant proportion of stroke patients in the Philippines arrive in the hospital emergency room late, often missing the crucial golden period for optimal treatment due to caretakers not recognizing unique presentations of stroke symptoms. Emphasizing the importance of public awareness campaigns in stroke recognition and management, city health offices should be reminded that early detection and treatment are essential in improving stroke outcomes. In the same way, early tackling of modifiable risk factors constitutes stroke prevention. In this article, we aim to call for action on two fronts outlined in the conceptual framework in Figure 1 to instigate a nationwide community-based stroke prevention program and to highlight the importance of decentralized public stroke awareness campaigns in local health communities, particularly in the context of the “new normal” brought about by the COVID-19 pandemic. The community-based stroke prevention program aims to identify and modify stroke risk factors within local communities, while the targeted stroke awareness campaigns focus on educating the public, more importantly the high-risk individuals along with their caretakers, about the manifestations and recognition of stroke. By addressing both aspects, we can tackle the multifaceted challenge of stroke prevention and management more effectively. We present a comprehensive approach that combines community-based stroke prevention with targeted stroke awareness campaigns, highlighting their complementary nature and the need for a multifaceted strategy to address the burden of stroke in the Philippines.

## 2. COVID-19, stroke, and the Philippine health system

Since 2020, the COVID-19 pandemic has greatly affected healthcare systems and people’s movement around the world. Lockdowns were imposed and public spending was diverted to support health infrastructures. Even though the World Health Organization (WHO) has recently already declared that the virus is no longer a public health emergency, (4) ironically, the Philippines hit a surge of cases in the same week as new variants arose. Back in 2020 and 2021, as ill patients overwhelmed the hospitals, many people were hesitant to seek health services for their medical concerns, especially those suffering from chronic lifestyle diseases to avoid being infected by the virus and thus, were lost to follow-up with their physicians. Furthermore, despite noted challenges in stroke care referral systems in the country and lack of delineation between primary care and specialist in low-resource settings (5), the pandemic has caused disruptions in healthcare access and utilization, particularly among lower-income individuals who may face financial barriers to seeking medical care. Since then, tertiary healthcare facilities have also tried to adapt to the challenges imposed by the pandemic. Acute stroke care algorithms were devised to be used in similar resource-constrained settings, providing valuable guidance for stroke management (6).

In a study in a Philippine tertiary hospital, during the pandemic, there was a notable decrease in stroke admissions, accompanied by delayed hospital consultation and a higher proportion of moderate to severe strokes (7). The discharge outcomes also indicated increased dependency and almost doubling of mortality rates (from 7 to 13%) among stroke patients compared to the pre-pandemic period (7). In addition, monitoring the performance of the health sector faced significant challenges due to the fragmented nature of health financing, the devolved structure of service delivery, and the presence of a mixed public-private health system. However, while this decentralized nature poses a challenge in implementing public health programs, we aim to use this as an opportunity regarding stroke awareness and prevention (8).

## 3. Community-based stroke prevention

While community health center stroke symptom awareness is an integral part of the proposed program, we acknowledge that attendance to this alone may not be sufficient to make an overall substantial impact on stroke prevention. A recent systematic review revealed that hypertension is still the frequently cited risk factor for stroke, along with diabetes, smoking, and elevated levels of cholesterol (9). Furthermore, in a large prospective cohort study of 155,722 individuals, approximately 70% of cardiovascular disease (defined as myocardial infarction, stroke and heart failure) cases, were found to be associated with modifiable risk factors (10). The authors recommend that health policies are made to target these identified risk factors to reduce mortality rates globally, considering different regions and populations (10). With these in mind, personalized risk factor assessments are recommended to be done at the local health centers to identify modifiable risk factors. This involves community health officers (physicians) with the help of local health workers screening in adults for elevated blood pressure, high cholesterol levels, uncontrolled diabetes, heavy smokers, and previous medical history of stroke so that patient education and interventions can be tailored depending on the individual’s risk profile. Therefore, our approach extends beyond symptom awareness to encompass the identification and modification of stroke risk factors through successful public health campaigns.

By integrating stroke risk factor identification, such as hypertension, diabetes, smoking, and elevated cholesterol levels, within community health centers, we can tailor patient education and interventions based on individual risk profiles. Additionally, this may also aid in the initial data collection if regional databases across the country are to be put up in the future. There is no national or regional database system in place hence, there is an existing difficulty in identifying high-risk individuals on a large scale. We recognize the importance of understanding the structure of health delivery in the Philippines to ensure the effective implementation of community-based stroke prevention strategies within local communities, considering the resources and healthcare access available to them.

## 4. Decentralized targeted stroke awareness

We also highlight the need for targeted awareness campaigns on stroke recognition to improve outcomes. Despite not having a unified national registry for stroke cases, the national stroke incidence rate ranged from 3.95 to 5.61%. [9] Under the framework of the building blocks of the health system by the WHO, an extensive review article by Collantes et al. highlighted the several gaps in stroke care in the Philippines (11). Among these is targeted improving patient awareness of stroke symptoms along with prompt emergency medical services (EMS) dispatch. Although efforts have been made to promote stroke awareness, it is evident that a significant lack of knowledge about stroke persists in different parts of the country (11). Mass media campaigns targeting the public for stroke awareness may have limited impact on behavior, highlighting the importance of well-designed and rigorously evaluated campaigns (12). Nonetheless, promoting awareness has become a key objective in several Asian countries since distinctive features found in Asian populations contribute to increased rates of stroke compared to Western populations (13).

Building on the risk factor assessment done as part of the two-pronged approach of the program, identified individuals, their family members, and caretakers will be prioritized for stroke education. In collaboration with local government leaders and healthcare practitioners, we recommend specifically targeting community education at the level of the local health center with a heavy emphasis on educating high-risk patients using different health promotion strategies for stroke recognition and prevention (14).

Engaging patients at risk in the Philippines can be facilitated through community health centers and *barangay* (local government unit) health workers. These health workers are already embedded in the communities and have direct access to the local population. Regular health check appointments can be scheduled in local health centers so that high risk individuals are followed up. One way to ensure retention is to use culturally adapted educational materials. Since the Philippine regions have varying cultures, dialects, and customs, strategies for dissemination and knowledge retention can be improved by making them more personalized. Educational materials are made to be culturally adapted and linguistically appropriate for different regions in the Philippines. This can include gender-specific materials, translating educational materials into local dialects, incorporating local customs and practices, and using culturally relevant examples to improve understanding and engagement. Patients and caretakers should also be able to show understanding of what was taught, and this must be ensured by the health professional educating.

As the country transitions into the new normal where long COVID-19 can potentially predispose entire populations to stroke, (15) information dissemination about preventive measures targeted toward the patient’s specific modifiable risk factors has increasingly become more important. Educating people about the signs, symptoms, and appropriate responses to a stroke patient can play a life-saving role. While further research is still needed in this area of stroke education, health promoters must be able to particularly adopt flexibility and this method has been seen to be partially effective (16). One example of a study done showed that gender-specific educational programs have a positive impact on stroke knowledge, particularly among women, indicating the need for targeted and tailored campaigns utilizing different media and educational messages for each gender (17).

Recent studies also suggest that COVID-19’s most common neurological manifestations in the Philippines are vascular disorders such as stroke, emphasizing the importance of raising stroke awareness in the context of the new normal (18–20). While there is no causality shown yet and variations in stroke presentations associated with COVID-19 have been observed across different regions and populations worldwide, it is crucial to understand the specific risks and thrombotic profiles relevant to the Philippines. By incorporating this understanding into our stroke awareness campaigns, we can deliver targeted messages to the public, empowering them with knowledge about stroke recognition, timely response, and the potential connection with COVID-19.

Despite advocating for a decentralized approach, a central directive at the level of the health department is also essential for the stroke awareness campaign. However, it needs to be personalized and very specific when it trickles down to the communities to be effective. Given the decentralized nature of the Philippine healthcare system, collaboration among national and local government units, health advocacy organizations, and health institutions is important. Establishing a stroke awareness coalition at both national and local levels can facilitate coordination and resource-sharing, optimizing the impact of the campaign.

Several outlets can be tapped to reach a wider network for the awareness campaign. Involving non-profit and non-government organizations such as the *Stroke Society of the Philippines* is a practical approach in the Philippines, where community-based organizations play a crucial role in healthcare initiatives. Partnering with them can help expand screening activities, especially in underserved areas. These organizations often have well-established networks already as well. Another one is providing talks in high schools and universities. Given the Philippines has a strong family-centric nature in communities, this may also help in disseminating knowledge about stroke risk factors. Educational institutions such as local public schools can serve as partners along with the health centers in the awareness campaign, with students becoming ambassadors of stroke prevention in their families and communities. To overcome concerns about attendance, our proposed strategy includes engaging the community through various channels, such as targeted media campaigns, and collaboration with local stakeholders, thereby increasing the reach and impact of the stroke awareness campaigns. Overall, this targeted approach aims to decrease the time gap before acting and ensure timely presentation to healthcare facilities for timely assessment and management, especially since there are only around 49 stroke-ready hospitals in the country (5).

## 5. Conclusion

Stroke remains a significant cause of mortality and disability in the Philippines, particularly among lower-income individuals who often experience delayed presentation and suboptimal treatment. The COVID-19 pandemic has further exacerbated the challenges in stroke diagnosis and management, necessitating a two-pronged approach involving community-based prevention and targeted public awareness campaigns. By integrating stroke risk factor assessment and modification within community health centers and at the same time, raising public awareness about stroke manifestations, we aim to reduce stroke incidence and improve outcomes of stroke patients. While there is a need for further groundwork research on the structure of Philippine health delivery system, particularly in the post-pandemic period to be able to implement these, our proposed strategies present an opportunity to make a significant impact on stroke awareness and prevention. With these, we aim to mitigate the burden of stroke and improve the overall health outcomes of Filipino stroke patients.

## Data availability statement

The original contributions presented in the study are included in the article/Supplementary material, further inquiries can be directed to the corresponding author.

## Author contributions

LVCS: Conceptualization, Formal analysis, Writing – original draft, Writing – review & editing. MCLG: Conceptualization, Writing – original draft.

## Conflict of interest

Author MCLG was employed by company ThinkWell Global.

The remaining author declares that the research was conducted in the absence of any commercial or financial relationships that could be construed as a potential conflict of interest.

## Publisher’s note

All claims expressed in this article are solely those of the authors and do not necessarily represent those of their affiliated organizations, or those of the publisher, the editors and the reviewers. Any product that may be evaluated in this article, or claim that may be made by its manufacturer, is not guaranteed or endorsed by the publisher.
